# Validation of a fully automated chemiluminescent immunoassay for cattle serum and plasma progesterone measurement

**DOI:** 10.3389/fvets.2022.1064201

**Published:** 2023-01-09

**Authors:** Ameer A. Megahed, Kristi L. Jones, Rafael S. Bisinotto, Ricardo C. Chebel, Klibs N. Galvão, Ann M. Chan, João H. J. Bittar

**Affiliations:** ^1^Department of Large Animal Clinical Sciences, College of Veterinary Medicine, University of Florida, Gainesville, FL, United States; ^2^Department of Animal Medicine (Internal Medicine), Faculty of Veterinary Medicine, Benha University, Moshtohor-Toukh, Kalyobiya, Egypt; ^3^Department of Research and Graduate Studies, College of Veterinary Medicine, University of Florida, Gainesville, FL, United States

**Keywords:** cattle, progesterone, immunoassay, validation, serum, plasma

## Abstract

**Introduction:**

Monitoring circulating progesterone concentration ([P4]) is an important component of basic and applied reproduction research and clinical settings. IMMULITE^®^ 2000 XPi (Siemens Healthineers, Cary, NC) is a newly upgraded fully automated immunoassay system marketed for human use to measure concentrations of different measurands including P4.

**Objectives:**

Our objective was therefore to characterize the analytical performance of the IMMULITE^®^ 2000 XPi P4 immunoassay (IPI) across the reportable range in serum and plasma of cattle.

**Methods:**

The IPI validation protocols included characterization of the method linearity, within-run, and between-run precision through calculation of the coefficient of variation (CV). The method accuracy was assessed through the calculation of the spiking-recovery (SR) bias across the reportable range (0.2–40.0 ng/mL). Passing–Bablok regression and Bland-Altman plots were used to determine the interlaboratory bias for two laboratories. Three types of observed total error (TEo) were calculated based on the considered type of bias, TEo_SR_ (spiking-recovery), TEo_RB_ (range-based bias), and TEo_AB_ (average-based bias).

**Results:**

The IPI was linearly related to the true value (*R*^2^ = 0.997) across the reportable range. The within-run and between-run precision (CV%) of the IPI for both serum and plasma [P4] of clinical relevance (1, 2, 5, and 10 ng/mL) were <5 and <10%, respectively. The TEo reported here for serum and plasma at [P4] of 1 and 5 ng/mL was ~20 and 25%, respectively. Of interest, the three types of TEo were relatively similar regardless of the considered bias.

**Conclusions:**

We concluded that the automated IPI provides a precise, accurate, reliable, and safe method for measuring [P4] in both serum and plasma of cattle. Consistent with the manufacturer's recommendations, the serum matrix is more accurate than plasma.

## Introduction

In the dairy and beef industries, good reproductive management is the main indicator of herd performance (1). Progesterone (P4) is a steroid hormone mainly synthesized and secreted from the corpus luteum (CL) in the ovary. Broadly speaking, P4 is the key hormone for regulating the normal cows' reproductive function through (1) regulation of the onset and the length of the estrus cycle, (2) facilitation of implantation and maintenance of pregnancy, (3) regulation of embryonic growth and development (2). Therefore, monitoring of circulating P4 concentrations ([P4]) is widely used as a key element for various clinical decisions related to the ovarian and uterine activity such as restoration of cyclicity after parturition, identification of ovarian cyst type, early pregnancy diagnosis, and prediction of parturition within 24 h with a threshold of plasma [P4] of 4.6 ng/mL (1, 3). The presence or absence of a functional CL with a threshold of serum [P4] of 1.0 ng/mL (4), or 0.3–0.5 ng/mL (5, 6), is often sufficient to be useful for clinical decisions in veterinary practice, management of reproduction, or classification of cows in a research study. The effective utilization of these known critical concentrations requires appropriate laboratory tests.

The selection of such medically appropriate laboratory tests requires knowledge about both clinical purposes and the analytical performance of the test. Therefore, researchers, clinicians, and laboratorians should collaborate in initiatives to improve test selection for clinical and/or research purposes. In the clinical laboratory setting, the analytical quality can be developed based on the concept of observed total analytic error (TEo) (7). This concept was introduced by Westgard and colleagues in 1974 (8), as a combination of imprecision (random error: variability between different measurements of the same thing) and bias (inaccuracy, or systematic error or bias: skewness of measurements from the true value). The analytical method can be considered adequate for clinical settings when TEo < allowable total error (TEa) based on the American Society for Veterinary Clinical Pathology guidelines (9). Allowable total analytic error (TEa) is the maximum analytical error we can tolerate and still detect clinically useful differences in results. It is determined through a clinical consensus discussion. Importantly, TEa is depend on several factors including, species, analyte concentration, clinical use, or type of laboratory (10). To date, the recommended TEa is not available for veterinary endocrinology (11). Therefore, calculated TEo can be used as a guide to determine the TEa for cattle P4 measured by IPI.

Several methods are available to measure circulating [P4] in cattle, including radioimmunoassay (RIA) which is considered the historical laboratory standard for measuring serum and plasma [P4] in cattle. However, this method is limited due to its inherent radiation hazard, equipment cost, decreased availability of reagents, and time-consuming (12). Therefore, a variety of enzyme-linked immunosorbent assays (ELISA), such as colorimetric ELISA read by absorbance spectrometers and chemiluminescent enzyme immunoassay (CLEIA) have been developed and evaluated to measure [P4] in cattle (13). Chemiluminescent immunoassay, which utilizes the generation of photons/light as a product of a chemical reaction, has become an attractive alternative to substitute RIA because it is safe for humans and the environment, easy to use, has a fast turnaround and has a high throughput (13). Siemens Healthineers has developed and marketed several series of IMMULITE^®^ systems using CLEIA technology including IMMULITE^®^ 1000, 2000, and 2000 XPi to measure concentrations of different measurands including P4 in humans. IMMULITE^®^ 2000 XPi is the newest model and is the only fully automated system that has been validated and optimized to accurately measure [P4] in human serum. To the best of our knowledge, there are no studies in bovine literature that have validated the IMMULITE^®^ 2000 XPi P4 immunoassay (IPI) for cattle; although, several veterinary diagnostic laboratories are using this technology to measure circulating blood [P4] in cattle (14). Therefore, there is a pressing need in both research and clinical settings for studies to validate the new system for cattle. Because the chemical structure, physiological mechanisms of P4, and serum components are conserved between humans and cattle, we hypothesized that the IPI will provide an accurate and reliable method for monitoring serum and plasma [P4] in cattle. The main aim of this study was therefore to characterize the analytical performance of the IPI for measuring serum and plasma [P4] in cattle.

## Materials and methods

All methods were approved by the University of Florida Institutional Animal Care and Use Committee (IACUC Study # 201810499, and Study # 201909630).

### Validation study outline

Seven of the nine immunoassay validation studies recommended by the guidelines of the Quality Assurance and Laboratory Standard committee of the ASVCP were used (9). Our approach was divided into three complementary phases (1) spike-recovery phase which included reportable range, within-run (repeatability), between-run (reproducibility), recovery, and detection limit studies; (2) interlaboratory comparison study, and (3) quality control rule validation study.

### Progesterone assay (Siemens)

The IPI kit, CLIA, is manufactured for use in Siemens Healthineers closed system IMMULITE^®^ 2000 XPi automated analyzer. The intended use per the manufacturer's product insert documentation is for *in vitro* diagnostics to measure serum [P4] in humans. The measuring range of the system is 0.2–40 ng/mL. Default settings report concentrations out of the calibrated range as < or > than the minimum or maximum concentration, respectively. The biologically relevant [P4] in cattle falls within this range therefore this default setting was utilized. Machine-specific verifiers and lot-specific adjustors were run following the manufacturer's recommended interval, 6 months and 2 weeks, respectively. All samples were analyzed using the same kit lot (Siemens Healthineers Inc., catalog number L2KPW2, lot # D585., Cary, NC) designed for IMMULITE^®^ 2000 XPi automated analyzer.

#### Spiking-recovery phase

Spiked serum and plasma (heparinized) samples were used for reportable range (linearity), repeatability (within-run precision), reproducibility (between-run precision), recovery, and limit of detection studies. Pooled serum and plasma samples were collected from 10 calves in order to obtain serum and plasma with [P4] as close as possible to zero. Serum and plasma samples were stored at −20° for a maximum of 48 h before use.

Commercially available certified reference material for P4 at a concentration of 1.0 mg/mL in acetonitrile (Cerilliant^®^ Sigma Inc., Round Rock, TX) was used as the source of P4. A working stock of P4 was made at 1,000 ng/mL and was then diluted to final spiked concentrations of 0.5, 1, 2, 5, 10, 15, 20, 30, and 40 ng/mL with the pooled serum, and 0.4, 0.7, 1, 2, 5, 7, 10, 15, 20, and 30 ng/mL with pooled plasma as the diluent matrix. Distilled water was used as a blank, and pooled serum (S_0_) and plasma (P_0_) matrices (non-spiked matrix) were the control. The range of spiked concentrations used correlates to the manufacturer's reported calibration range, 0.2–40 ng/mL. All measurements using spiked samples used five replicates each day for five consecutive days. Samples were stored at −20°C between each consecutive analysis.

##### Reportable range study (linearity)

Five within-run replicates of each spiking concentration were performed on day 1. Averages for each concentration were calculated. The association between measured means and the spiked concentrations was assessed using a scatter plot and simple linear regression was performed. Paired *t*-test was used for a rough estimation of the difference between measured concentration and spike concentration data.

##### Within-run (repeatability) study

To test the repeatability of the system across the reportable range, five within-run replicates from each spiking level were used. The average coefficient of variation percentages (CV%) was calculated at each level and plotted against the spiked concentrations, and trend lines were generated.

##### Between-run (reproducibility) study

The averages of five replicates for each spiking concentration over five consecutive days were used to test the reproducibility of the system. The average CV% was calculated and plotted against the spiked concentrations and trend lines were generated.

##### Recovery study

The recovery percentage was calculated for each spiked concentration (denoted “S_x_ or P_x_”) as:


$$\begin{array}{l} \text{Recovery}\left(\%\right)=\left[\left({\text{Sx}}_{\text{recoverd}}-{\text{S}}_{0}\right)/{\text{Sx}}_{\text{spiked}}\right]\;\times \;100\\ \text{Recovery}\left(\%\right)=\left[\left({\text{Px}}_{\text{recoverd}}-{\text{P}}_{0}\right)/{\text{Px}}_{\text{spiked}}\right]\;\times \;100 \end{array}$$

The Spiking-recovery bias percentage (SRB) was then calculated using the formula:


$$\begin{array}{l} \text{SRB}\left(\%\right)=\text{Recovery}\%-100 \end{array}$$

The SRB for each spiked concentration was plotted on a function graph with SRB (*y*-axis) against spiked concentrations (*x*-axis).

##### Detection limit study

We conducted the detection limit study to verify the manufacturer's decision of defining 0.2 ng/mL as the lower limit. The limit of blank (LOB) which determines the highest measurable [P4] in the blank was calculated using the following formula:


$$\begin{array}{l} {\text{LOB}=\text{Blank}}_{\text{mean}}+1.65\;\times \text{ SD} \end{array}$$

The limit of detection (LOD), which determines the lowest amount of [P4] in the sample which can be detected but not necessarily quantified as an exact value or the smallest [P4] in the test sample that we can easily distinguish from zero was calculated using the following formula:


$$\begin{array}{l} \text{LOD}={\text{S}}_{0-\text{mean}}+1.65\;\times \text{ SD}\\ \text{LOD}={\text{P}}_{0-\text{mean}}+1.65\;\times \text{ SD} \end{array}$$

The limit of Quantification (LOQ), the smallest concentration of P4 in the test sample that we can determine with acceptable precision and accuracy, was also calculated using the following formula:


$$\begin{array}{l} \text{LOQ}={\text{S}}_{\text{x}-\text{mean}}+2\;\times \text{ SD}\\ \text{LOQ}={\text{P}}_{\text{x}-\text{mean}}+2\;\times \text{ SD} \end{array}$$

#### Interlaboratory comparison study

To assess the average bias (AB), and the range-based bias (RB) across various concentrations between two laboratories, an interlaboratory comparison study was performed using 40 plasma samples collected from 40 Holstein-Friesian heifers treated with two different analogs and doses of prostaglandin-F2α (15). We used plasma samples because the system error (random, systematic, and total errors) was higher in plasma than in serum. Plasma [P4] was measured in our laboratory using IPI at the College of Veterinary Medicine, University of Florida (UF). Samples were then sent out overnight to the reference laboratory at the Endocrinology Diagnostic Laboratory, College of Veterinary Medicine, University of Tennessee (TN) to measure [P4] using the IMMULITE^®^ 2000 XPi system. The plasma samples were selected to represent the normal biological range of [P4] in cattle.

Passing-Bablok regression analysis was used to identify the line of best fit. In Passing-Bablock regression, the intercept value reflects constant bias, and the slope value reflects proportional bias. Constant and proportional biases were considered significant when the 95% confidence intervals (CI) did not include zero and one, respectively.

Bland-Altman plots were constructed to characterize the agreement between the two laboratories. The mean bias and percentage bias, and the associated 95% CI were calculated (16). The mean constant bias describes the differences between the two laboratory analyzers being consistently above or below 0. Percent bias describes the increase or decrease in the difference between the two laboratories' readings in proportion to a relative increase in the mean value. Linear regression was used to assess the proportional error in Bland-Altman plots (16, 17). Upper and lower limits of agreement (LoA) were calculated using the following equation:


$$\begin{array}{l} \text{Mean bias}\pm 1.96\times \text{SD} \end{array}$$

The plot was visually examined to determine if the differences were symmetrically distributed around 0 (homoscedastic) and that 95% of the differences were between the upper and lower LoA (18). The IPI system was appropriate for measuring [P4] in the sample matrix when the 95% CI for LoA < TEa (19). Statistical analyses were performed using MedCalc Statistical Software version 20.110 (MedCalc Software bvba. Ostend, Belgium, 2018), R Studio version 4.1.3, and SAS^®^ OnDemand for Academics (PROC HPSPLIT; SAS Inst. Inc., Cary, NC). Statistical significance was set at *P* < 0.05.

### IMMULITE^®^ 2000 XPi P4 assay total error computation

The observed total error percentage of IMMULITE^®^ system was calculated using the following formula:


$$\begin{array}{l} \text{TEo }\left(\text{\%}\right)=2\text{CV\%}+\text{absolute value of bias\%} \end{array}$$

Bias (%) = (Average absolute deviation from the target value/target value) × 100

According to the type of calculated bias, three types of TEo were calculated:

1) TEo_SR_ (spiking-recovery bias) as follows
  $$\begin{array}{l} {\text{TEo}}_{\text{SR }}\left(\%\right)=2\text{CV\%}\left(\text{within }-\text{ run}\right)+\text{absolute SRB\%} \end{array}$$

The 40 plasma samples used for the comparison study were divided into five different concentration ranges of eight samples each (group 1: 0.2–1.5; group 2: 1.8–2.9; group 3: 4.0–5.4; group 4: 4.8–8.8; and group 5: 10.2–25.8 μg/dL);

2) TEo_RB_ was calculated using the difference bias % from the Bland-Altman plot of each group and the within-run CV% of correspondence spiking concentration as follows:
  $$\begin{array}{l} {\text{TEo}}_{\text{RB}}\left(\%\right)=2\text{CV\%}+\text{absolute difference bias\%} \end{array}$$
3) TEo_AB_ (average-based bias) was calculated using the total average difference bias% from the Bland-Altman plot of the comparison study and the within-run CV% as follows:
  $$\begin{array}{l} {\text{TEo}}_{\text{AB}}\left(\%\right)=2\text{CV\%}+\text{absolute difference bias\%} \end{array}$$

#### Quality control validation study

Three commercially available levels (0.64, 7.5, and 20.9 ng/mL) of quality control (QC) samples (Lyphocheck, Bio-Rad Inc., Hercules, CA) were used as system quality assurance each day in accordance with the manufacturer's recommendations. Five replicates of each QC level were run for five consecutive days. Within-run and between-run CV% was calculated for each level.

## Results

### Spiking-recovery phase

#### Progesterone concentration in serum and plasma matrices

The average [P4] in the pooled serum matrix were 0.28, <0.20 (below detectable limit), <0.20, 0.21, and 0.26 ng/mL in days 1, 2, 3, 4, and 5, respectively. In the pooled plasma matrix, the average [P4] in days 1, 2, 3, 4, and 5 were 0.28, 0.28, 0.30, 0.35, and 0.32 ng/mL, respectively. The plasma matrix [P4] tested at the external testing facility (TN) was 0.27 ng/mL.

#### Linearity

**Figure 1:**
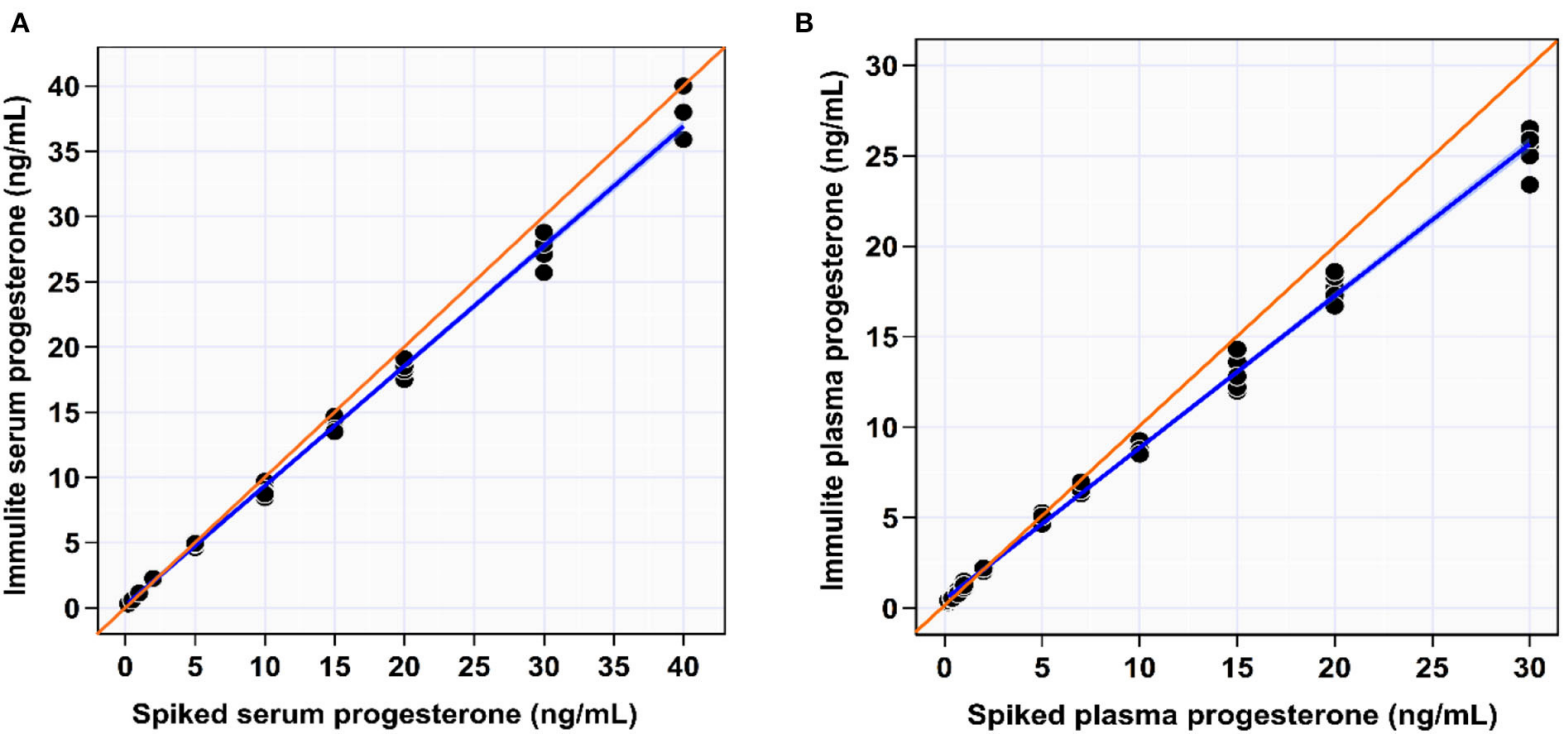
Scatter plot of spiked serum **(A)** and plasma **(B)** progesterone concentrations measured by IMMULITE^®^ 2000 XPi P4 immunoassay with blue regression line and confidence bands for the regression lines. The identity line is orange. Regression line equation for spiked serum samples: true [P4] = −0.16 + 1.06 × [P4]_IMMULITE_; Regression line equation for spiked plasma samples: true [P4] = −0.50 + 1.18 × [P4]_IMMULITE_.

The IPI showed an excellent linear association with the spiked serum (*R*^2^ = 0.997) and plasma samples (*R*^2^ = 0.996) over the reportable range. The linear regression of spiked serum samples showed an intercept of −0.16 ng/mL (close to 0; *P* = 0.327) and a slope of 1.08 ng/mL (*P* < 0.001), indicating the absence of constant bias and the presence of proportional difference (Supplementary Table S1; Figure 1A). The paired *t*-test for linearity indicates a significant deviation from linearity (*P* < 0.001).

The intercept and slope of the linear regression of spiked plasma samples were −0.5 ng/mL (*P* < 0.001) and 1.2 ng/mL (*P* < 0.001), respectively, indicating the presence of constant and proportional biases (Table 1; Figure 1B). The paired *t*-test for linearity indicates a significant deviation from linearity (*P* < 0.001). However, the IPI seems to correctly measure [P4] up to 5 ng/mL in both serum and spiked plasma samples before starting to underestimate the [P4].

**Table 1:** Coefficient of variation (CV; within-run), bias, and observed total error (TEo) percentages results of IMMULITE^®^ 2000 XPi P4 immunoassay across the spiked plasma progesterone concentrations.

**Plasma [P4]**			**Precision %**	**Bias (%)**			**TEo (%)**		

**Plasma**	**Spiked P4**	**[P4] (ng/mL)**	**CV% (within-run)**	**Spiking-recovery (SR)**	**Range-bases (RB)**	**Average-based (AB)**	**TEo**_SR_	**TEo**_RB_	**TEo**_AB_
L1	0.4	0.6	12.0	−40.9	19.1	9.9	64.9	43.1	33.9
L2	0.7	0.9	7.7	−22.5	19.1	9.9	37.8	34.5	15.4
L3	1.0	1.3	9.8	−7.2	19.1	9.9	26.8	38.7	19.6
L4	2.0	2.2	4.5	−9.0	7.3	9.9	18.0	16.3	9
L5	5.0	5.0	5.2	−7.6	5.0	9.9	18.0	15.4	10.4
L6	7.0	6.6	3.7	−10.3	9.2	9.9	17.6	16.6	7.4
L7	10.0	8.9	3.2	−14.8	8.3	9.9	21.1	14.7	6.4
L8	15.0	13.0	7.4	−15.8	8.3	9.9	30.7	23.1	14.8
L9	20.0	17.7	4.3	−13.2	8.3	9.9	21.8	16.9	8.6
L10	30.0	25.3	4.7	−16.9	8.3	9.9	26.3	17.7	9.4

#### Within-run precision (repeatability) study

**Figure 2:**
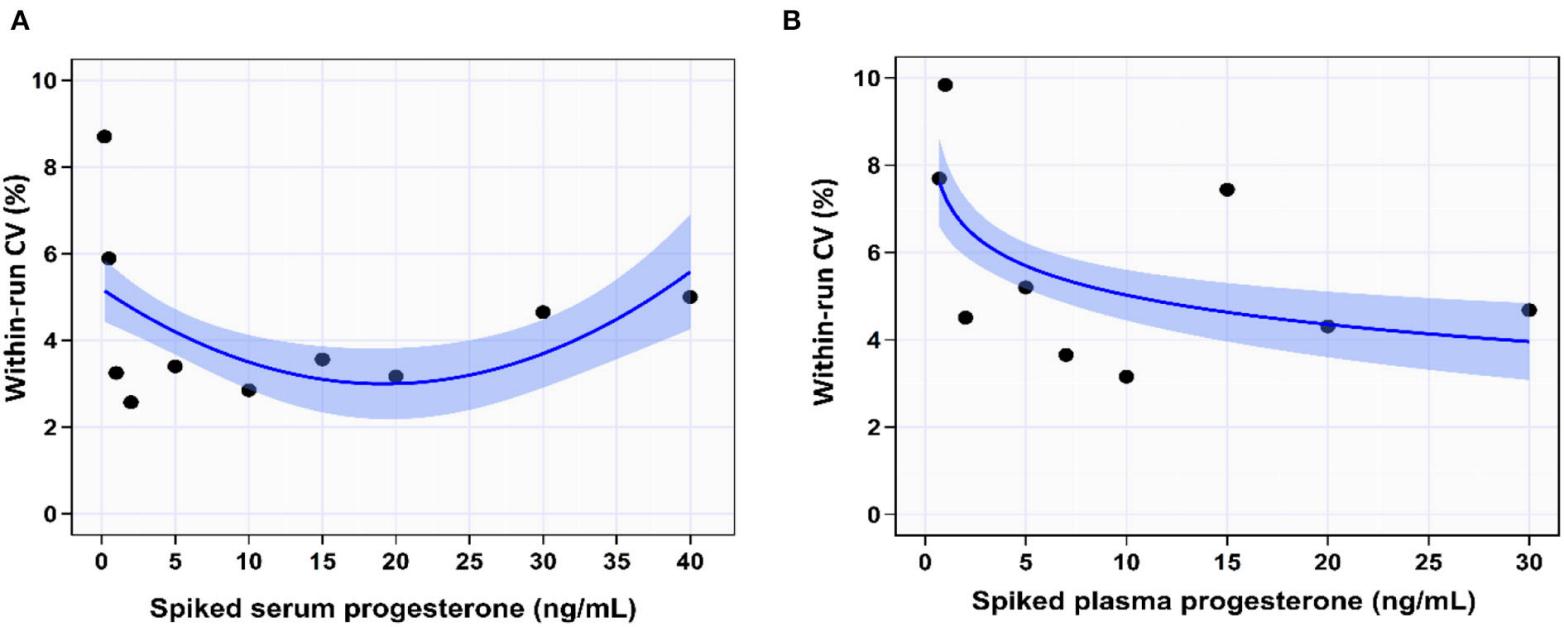
The coefficient of variation percentage (CV%) indicated the within-run precision of IMMULITE^®^ 2000 XPi P4 immunoassay across the spiked serum **(A)** and plasma **(B)** progesterone concentrations. The solid blue lines are the best-fit regression lines. The light blue shaded areas indicate the 95% confidence bands for the regression lines.

In the spiked serum samples, the IPI imprecision (within-run CV%) decreased with increasing serum [P4] up to 20 ng/mL then it started to increase slightly with increasing serum [P4] (trendline: quadratic function, CV% = 5.2 – 0.2 × [P4] + 0.01 × [P4]^2^) as shown in Supplementary Table S1 and Figure 2A. It increased from 4.7% at serum [P4] of 30 ng/mL to 3.3% at serum [P4] of 1.0 ng/mL. The overall within-run impression of the IPI in the serum samples was 4.3%.

In the spiked plasma samples, the imprecision increased with decreasing plasma [P4] (trendline: power function, CV% = 8.9 – 1.7 × log[P4]), as shown in Table 1 and Figure 2B. It increased from 4.6% at plasma [P4] of 30 ng/mL to 9.8% at plasma [P4] of 1.0 ng/mL. The overall within-run impression of the IPI in the plasma samples was 6.2%.

#### Between-run precision (reproducibility) study

**Figure 3:**
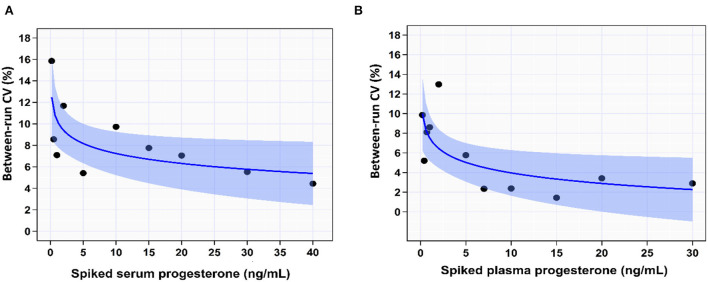
The coefficient of variation percentage (CV%) indicated the between-run precision of IMMULITE^®^ 2000 XPi P4 immunoassay across the spiked serum **(A)** and plasma **(B)** progesterone concentrations. The solid blue lines are the best-fit regression lines. The light blue shaded areas indicate the 95% confidence bands for the regression lines.

In the spiked serum samples, the between-run imprecision (CV%) increased with decreasing serum [P4]. The shift of the curve occurred at serum [P4] of 5.0 ng/mL, where the imprecision starts to decrease slightly with increasing serum [P4] (trendline: power function, CV% = 10.3 – 1.3 × log[P4]). It increased from 5.5% at serum [P4] of 30 ng/mL to 7.1% at [P4] of 1.0 ng/mL. The overall between-run impression of the IPI in the serum samples was 8.3% (Supplementary Table S1; Figure 3A).

In the spiked plasma samples, the imprecision increased with decreasing plasma [P4]. Similar to the spiked serum sample, the shift of the curve occurred at serum [P4] of 5.0 ng/mL, where the imprecision starts to decrease slightly with increasing serum [P4] (trendline: power function, CV% = 7.5 – 1.5 × log[P4]). It increased from 2.9% at plasma [P4] of 30 ng/mL to 8.6% at plasma [P4] of 1.0 ng/mL. The overall between-run impression of the IPI in the plasma samples was 5.7% (Figure 3B).

#### Recovery study

**Figure 4:**
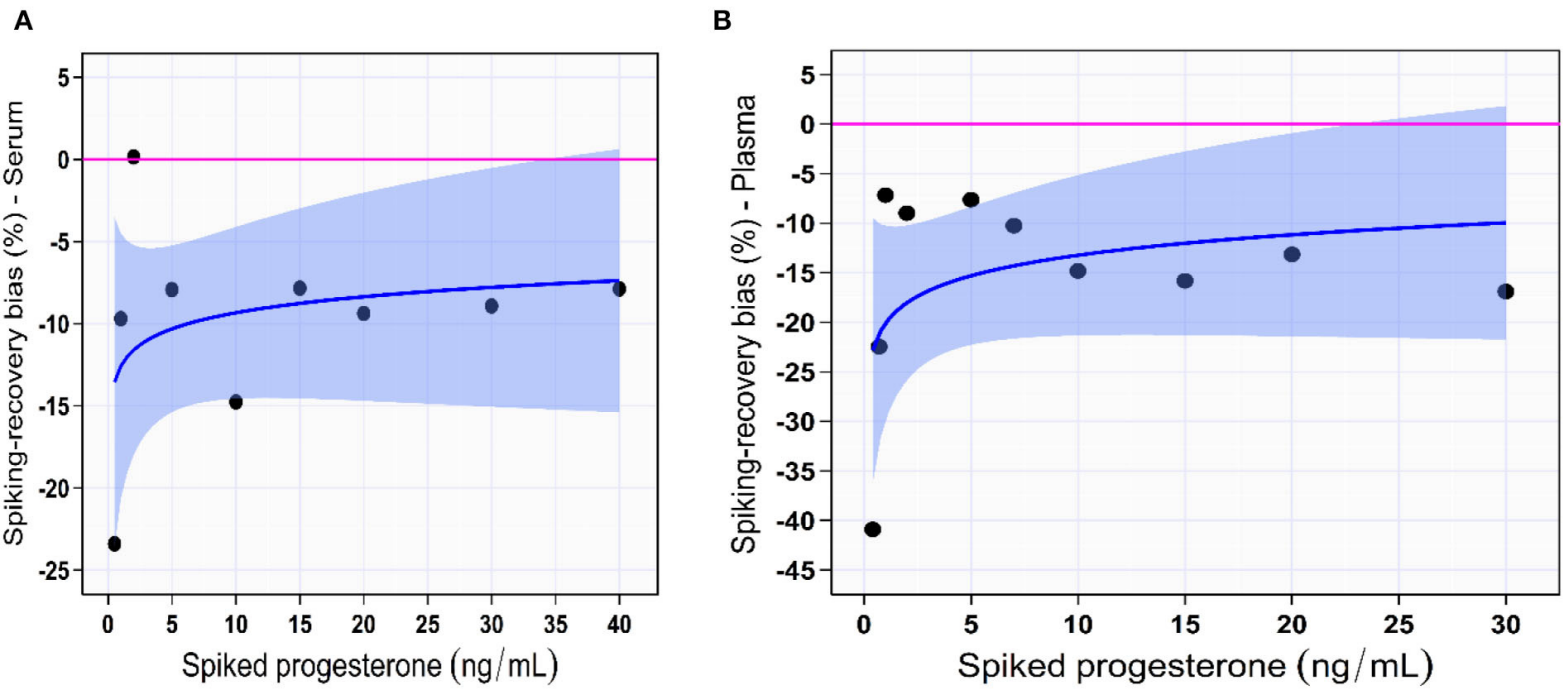
Spiking-recovery bias percentage of IMMULITE^®^ 2000 XPi P4 immunoassay across the spiked serum **(A)** and plasma **(B)** progesterone concentrations. The solid blue line is the best-fit regression line. The solid blue lines are the best-fit regression lines. The light blue shaded areas indicate the 95% confidence bands for the regression lines.

The absolute SRB was the highest at [P4] < 1.0 ng/mL in both serum (23.4%, [P4] of 0.5 ng/mL) and plasma (40.9%, [P4] of 0.4 ng/mL) samples. However, The SRB was minimal (< 10%) for spiked serum [P4] > 1.0 ng/mL (trendline: logarithmic; SRB [%] = −12.6 + 1.5 × log[P4]; Supplementary Table S1; Figure 4A). In the spiked plasma samples, The SRB was minimal (<10%) for [P4] ranging from 1.0 to 5.0 ng/mL, then starts to increase reaching 16.9% for [P4] of 30 ng/mL (trendline: logarithmic; SRB% = −20.7 + 3.3 × log[P4]; Table 1; Figure 4B).

#### Detection limit study

The LOB was undetectable (<0.2 ng/mL) in both serum and plasma matrices, indicating that the manufacturer's decision to define 0.2 ng/mL is adequate. The LOD in the serum matrix was 0.32 ng/mL; however, in the plasma matrix LOD was 0.43 ng/mL. The LOQ determined on the spiked serum sample of [P4] of 1.0 ng/mL was 1.26 ng/mL with a corresponding between-run CV of 8.6%, 10 ng/mL was 9.3 ng/mL with a corresponding between-run CV of 9.7%, and 30 ng/mL was 30.2 ng/mL with a corresponding CV of 5.5%.

In the spiked plasma samples, the LOQ determined on the spiked plasma sample of [P4] of 1.0 ng/mL was 1.5 ng/mL with a corresponding between-run CV of 8.6%, 10.0 ng/mL was 9.4 ng/mL with a corresponding CV of 2.4%, and 30.0 ng/mL was 27.6 ng/mL with a corresponding CV of 2.9%.

### Inter-laboratory method comparison study

**Figure 5:**
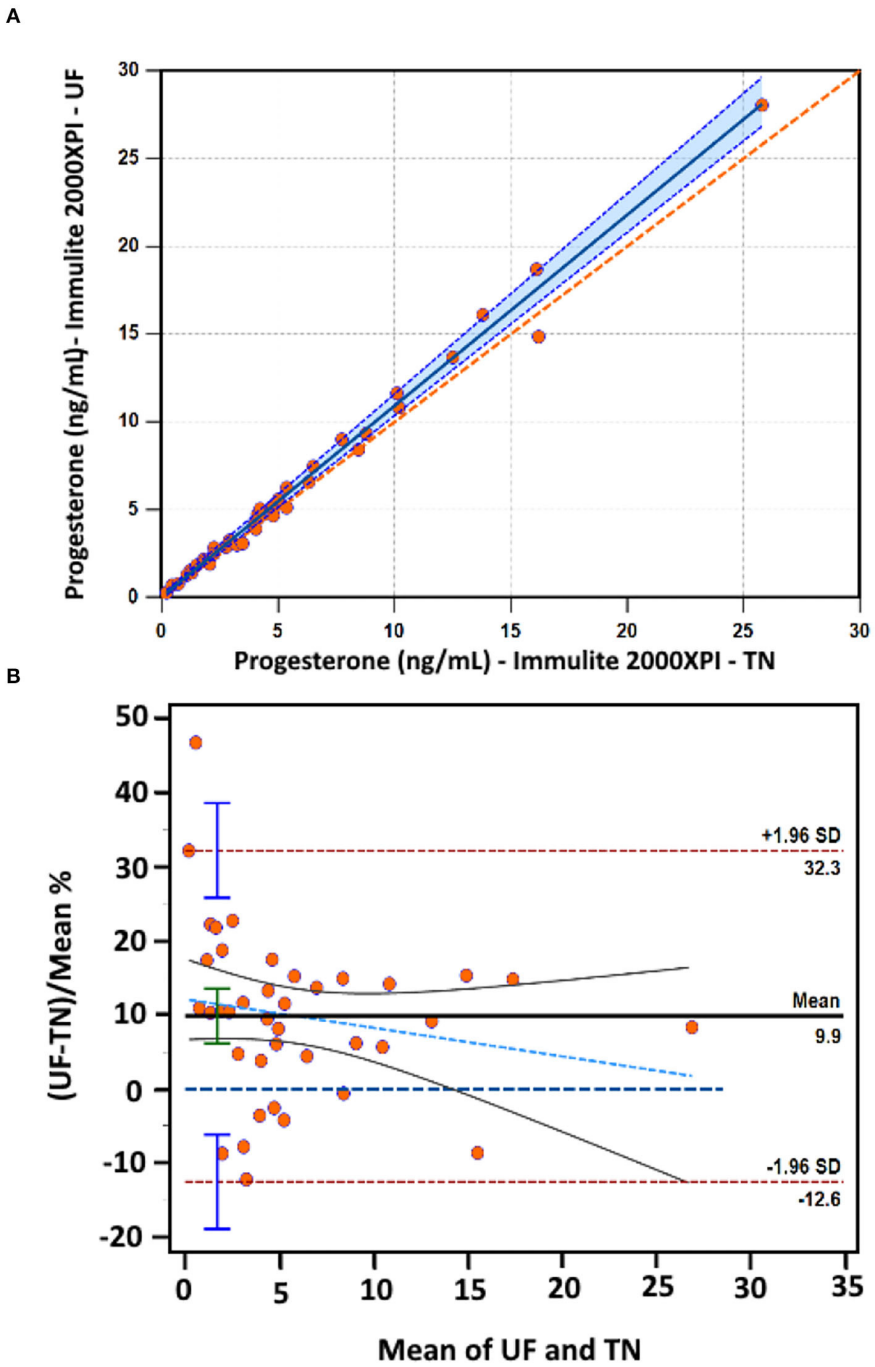
**(A)** Scatterplot indicating the relationship between the plasma progesterone concentration ([P4]) measured by the IMMULITE^®^ 2000 XPi P4 immunoassay at the University of Florida (UF) and at the University of Tennessee (TN). The orange dashed diagonal line is the line of identity, and the solid blue line is the line of best fit from Passing-Bablok, and the light blue shaded area is the 95% confidence interval (CI). **(B)** Bland-Altman mean percentage difference plot. The horizontal black solid line represents the mean bias percentage and the horizontal maroon long dashed lines reflect the 95% limits of agreement. The horizontal solid blue long dashed line represents the line of identity. The light blue long dashed line and surrounding gray solid lines represent the regression line and the 95% CI, respectively. The vertical blue and green bars indicate the 95% confidence interval.

An adequate linear relationship existed between plasma [P4] measured by IPI at UF and TN laboratories. Passing Bablock regression indicated presence of proportional bias (slope = 1.09; 95% CI, 1.04–1.14) and absence of constant bias (intercept = 0.05 ng/mL; 95% CI, −0.07 to 0.19) (Figure 5A).

The Bland-Altman plot indicated that the IPI at UF measured plasma [P4] higher than the IPI at TN with 0.47 ng/mL (95% CI, −1.0 to 1.9 ng/mL; *P* < 0.001; Supplementary Figure S1), and percentage bias of 9.9% (95% CI, 6.1–13.6 %; *P* < 0.001). The 95% LoA were from −12.6% (95% CI, −19.0 to −6.2) to 32.3% (95% CI, 25.9–38.7). Inspection of the plot indicated that the differences were homoscedastic, and ~95% of the data points were within the LoA (Figure 5B). The proportional error was not present in the plot based on the estimated slope value for linear regression of differences against mean values (slope = −0.39; *P* = 0.26). The 95% CI for LoA, representing the difference between −12.6 and 32.3%, was 44.9%.

#### IMMULITE^®^ 2000 XPi P4 assay total error computation

**Figure 6:**
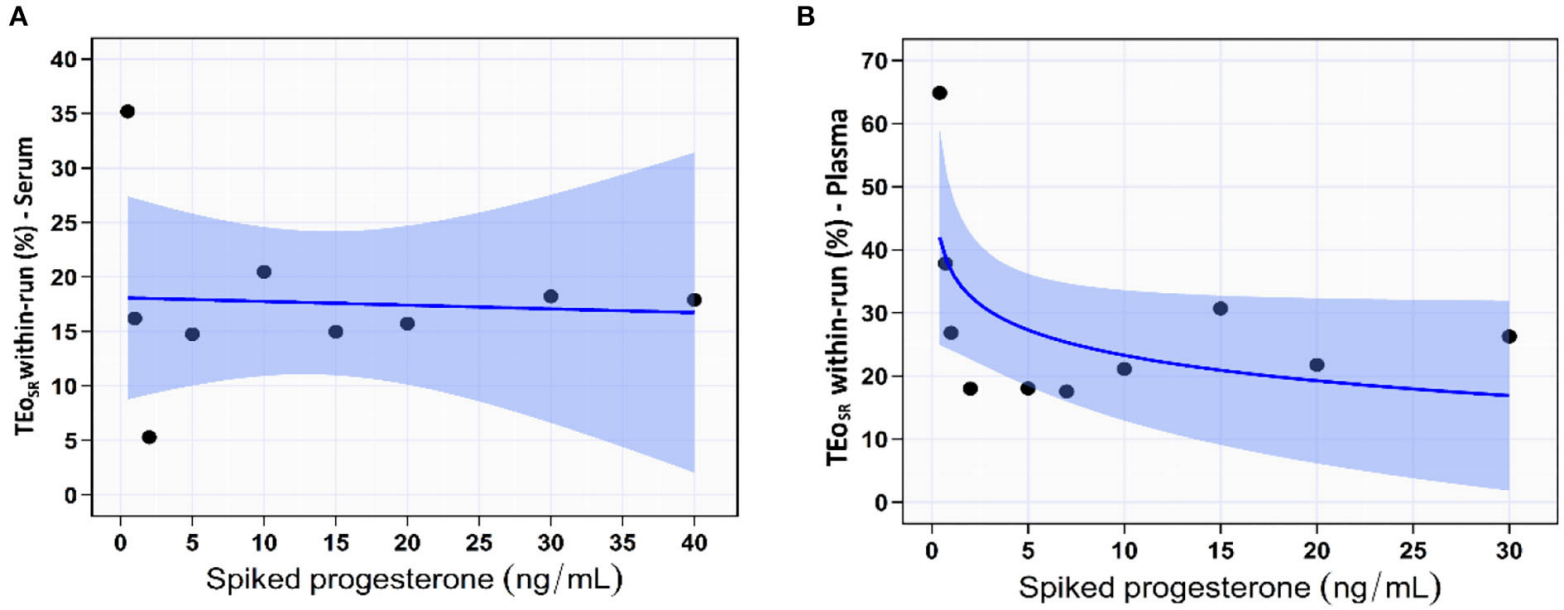
Observed total error percentage (TEo) of IMMULITE^®^ 2000 XPi Progesterone immunoassay across the spiked serum **(A)** and plasma **(B)** progesterone concentrations. The solid blue lines are the best-fit regression lines. The light blue shaded areas indicate the 95% confidence bands for the regression lines.

The TEo_SR_ was relatively constant over the spiked serum [P4] (slope = −0.11; *P* = 0.876) with an average of 15.4% for [P4] ranging from 1.0 to 40.0 ng/mL; however, TEo_SR_ was 35.2% at S1 (0.5 ng/mL; Supplementary Table S1; Figure 6A). In the spiked plasma samples, TEo_SR_ seems to increase with decreasing in [P4] (trendline: logarithmic; TEo_SR_% = 36.7 – 5.8 × log[P4]), with an average 24.2% for [P4] ranging from 0.5 to 30 ng/mL; however, TEo_SR_ was 64.9% for P1 (0.4 ng/mL; Table 1; Figure 6B). Interestingly, the TEo_SR_, TEo_RB_, and TEo_AB_ across the spiked plasma [P4] were relatively similar (Table 1).

### Quality control validation study

The within-run imprecision of QC was higher for level 1 of 0.64 ng/mL ([P4] = 0.7 ng/mL; CV = 8.8%) than level 2 of 7.5 ng/mL ([P4] = 8.8 ng/mL; CV = 2.7%) and level 3 of 20.9 ng/mL ([P4] = 22.2 ng/mL; CV = 2.9%).

Similarly, the between-run imprecision was higher for level 1 ([P4] = 0.8 ng/mL; CV = 6.3%) than the level 2 ([P4] = 8.5 ng/mL; CV = 1.1%) and level 3 ([P4] = 23.1 ng/mL; CV = 3.0%). The bias and TEo of QC were minimal for level 1 (7.7 and 20.4%, respectively), and level 2 (2.6 and 4.7%, respectively). and level 3 (6.6 and 12.6%, respectively) indicating the desirable stability properties.

## Discussion

Measuring circulating [P4] in cattle is limited to research settings because verified methods, primarily RIA, are time-consuming, expensive, have hazards involved, and are phasing away. Therefore, validation of fully automated IPI for cattle can increase the use of circulation [P4] testing in clinical settings; therefore, it benefits management practices. To the best of our knowledge, this study is the first study to comprehensively validate cattle serum and plasma [P4] measured by the IPI over a measurable range of 0.2–40 ng/mL. Although two previous reports evaluated the analytical performance of IMMULITE^®^ 1000 P4 immunoassay in cattle (20, 21), neither of these studies investigated the between-run precision (CV), accuracy (bias), and TEo of the immunoassay nor used plasma. Therefore, the main strength of the data reported here lies in the characterization of the IPI precision (CV), bias, and TEo across the entire reportable range. The results of this study showed that the manufacturer's default protocol of the automated IMMULITE^®^ 2000 XPi system is capable of accurately measuring [P4] in both cattle serum and plasma samples.

The manufacturer's reportable [P4] measurement range of the IPI is 0.2–40 ng/mL. This means that the system can measure [P4] from 0.2 to 40 ng/mL with acceptable precision and accuracy. The reportable range of the immunoassay was evaluated by linearity. The automated IPI showed adequate linearity in both serum and plasma confirming that the reportable range provided by the manufacturer is achievable in both cattle serum and plasma samples. Interestingly, this reportable range is in line with the biological circulating [P4] range (0.1–30.0 ng/mL) in cattle (3, 22). Unlike RIA, IMMULITE^®^ 2000 XPi has an inaccessible calibration curve in the software, and two adjustors are used to adjust the calibration curve. Circulating [P4] measured outside the Immulite measurement range is reported as <0.2 or >40.0 ng/mL. However, the calibration verifier mode (CVM) and the range change software (RCS) are two options provided by IMMULITE^®^ 2000 XPi to measure samples with [P4] outside the manufacturer's measuring range. The CVM eliminates the limits of the measurable range; however, it is not used in veterinary or human medicine. It is typically used for linearity testing to verify the reportable range every 6 months as required by most accrediting laboratories. The RCS is an exclusive software provided by Siemens to veterinary diagnostics laboratories to be able to set up new reportable ranges for each measurand each new lot, but It is the responsibility of the diagnostic laboratory to verify and document the acceptability of the new measuring range (IMMULITE^®^ 2000/2500 Operator's Manual 2007).

Based on the ASVCP guidelines, the repeatability and reproducibility should not exceed ~5 and 10% (25 and 33% of TEa), respectively, which is easily achievable in biochemistry measurands (9). However, IPI has lower precision, therefore CV% may exceed this recommended limit. In this study, IPI showed excellent repeatability (CV < 5%) and reproducibility (CV < 10%) in both serum and plasma samples with [P4] above 1.0 ng/mL, and acceptable repeatability in both serum and plasma samples with [P4] < 1.0 ng/mL. Therefore, the automated system is precise enough to be used in research and clinical settings for measuring serum and plasma [P4] in cattle. Taking an example of [P4] of clinical relevance (1.0 ng/mL, indicating ovarian luteal activity), IMMULITE^®^ showed a between-run CV of nearly 10%. This means that samples with exactly [P4] of 1.0 ng/mL have a 95% probability of being measured within 1.0 ng/mL ± 2 SD which is 0.92–1.08 ng/mL. Interestingly, any value within this range does not significantly affect the clinical decision. The precision of IMMULITE^®^ 2000 XPi reported in this study is consistent with earlier findings (20, 21). Martin and colleagues in 2007 reported within-run precision (IMMULITE^®^ 1000) of 8.0, 13.9, 1.1, 7.8, and 2.2% for the serum blank and the samples spiked with 0.25, 1, 5, and 25 ng/mL of added P4, respectively (20). However, Reis et al. in 2015 reported an average within-run CV (IMMULITE^®^ 1000) of 6.03% for plasma spiked with 0.0, 0.5, 1, 2.5, 5, and 10 ng/ml of added P4 (21).

It is important to mention that the precision evaluation of any analytical methods alone will not account for the difference in analytical performance between methods or laboratories. Therefore, bias needs to be addressed for the interpretation of the results. The Bland-Altman showed a minimal percentage bias between the two laboratories of 9.9% over plasma [P4] range 0.28–28.1 ng/mL. Taking an example of [P4] of clinical relevance (1.0 ng/mL), IMMULITE^®^ in our facility (UF) measures plasma [P4] 0.1 ng/mL higher than the reference laboratory (TN), indicating a minimal positive bias in our facility with no clinical significance. However, the visual analysis of the Bland-Altman graph (Supplementary Figure S1) showed that the mean bias increased with increasing plasma [P4].

An additional strength of this study is the calculation of three types of biases (SR, RB, and AB) and three types of TEo (TEo_SR_, TEo_RB_, or TEo_AB_). Each type of bias has different properties and provides a certain type of information. For example, SR bias reflects the interior bias of the IPI over the spiked [P4] range, RB and AB show the bias between the two laboratories. Range-based bias is considered the most relevant type of bias in the clinical setting because it shows the bias between two laboratories at a given [P4], but the AB averages all RB biases which minimizes the true bias (7). The observed total error refers to the analytical variability of the results, which is considered one aspect of several that should be taken into account for test results interpretation (9). The average TEo of IPI reported in this study was ~30%. This means that samples with exactly [P4] of 1.0 ng/mL, have a 95% probability of being measured within 1.0 ng/mL ± TEo which is 0.8–1.2 ng/mL. From the perspective of clinical importance, this percentage of the total error of the automated IPI is acceptable and consistent with the recommendations of ASVCP guidelines (2019) and the immunoassay guidance manual (23).

Despite the advantages of plasma which include large volume, no delayed clotting, and less risk of hemolysis, the results of this study showed that serum is a more sensitive matrix than heparinized plasma for measuring [P4] using IPI in agreement with the manufacturer's recommendations. This is might because of the influence of anticoagulants on the assay, the protein binding capacity, and the stability of the sample. Among pre-analytical factors, little is known regarding the effects of anticoagulants on the analytical performance of immunoassays (24). However, using EDTA as an anticoagulant has been shown to have a significant impact on the analytical performance of immunoassays, resulting in poor agreement between serum and plasma for the measurement of several hormones in dogs (25, 26). Additionally, serum collection includes the removal of fibrinogen, platelets, and other circulating proteins that could interact with immunoassay reagents (27). The main limitation of this study is that the clinical performance of the automated system has not been investigated because our main goal was to calculate the random, systematic, and total errors over the reportable range. Additionally, we didn't investigate the effects of hemolysis and lipemia as interference on the analytical performance of the system; therefore, future research is needed.

## Conclusions

The new fully automated IPI provides a precise, accurate, and reliable safe method for measuring [P4] in the serum and plasma of cattle. Serum is a more accurate matrix compared to plasma in measuring circulating [P4] using IPI as recommended by the manufacturer. This study provides important information about the precision and accuracy of IPI that should be considered in the interpretation of results and for future expert consensus discussions to determine the recommendations for allowable total error (TEa).

## Data availability statement

The datasets presented in this article are not readily available because a further validation study using this data set is undergoing. Requests to access the datasets should be directed to JB, jbittar@ufl.edu.

## Ethics statement

The animal study was reviewed and approved by University of Florida Institutional Animal Care and Use Committee.

## Author contributions

AM, KJ, and JB conceived and designed the study, analyzed the data, and drafted the manuscript. AM, KJ, RB, RC, AC, and JB contributed to data collection and unit maintenance. AM, KG, and JB wrote the manuscript. All authors have read and agreed to the published version of the manuscript.
