# Supplementary material for: Validation of a fully automated chemiluminescent immunoassay for cattle serum and plasma progesterone measurement
Source: Front Vet Sci. 2023 Jan 9;9:1064201. doi: 10.3389/fvets.2022.1064201 (PMC9868466; doi:10.3389/fvets.2022.1064201)

**Table S1.** Coefficient of variation (CV; within-run, between-run), spiking-recovery bias, and observed total error (TEo) percentage results of IMMULITE^®^ 2000 XPi P4 immunoassay across the spiked serum progesterone concentrations.

| Serum [P4] | | | Precision % | | Bias (%) | TEo (%) |
| --- | --- | --- | --- | --- | --- | --- |
| Level | Spiked P4 | [P4] (ng/mL) | CV (within-run) | CV (Between-run) | Spiking-recovery (SR) | TEo_SR_ |
| L2 | 0.5 | 0.7 | 5.9 | 8.6 | -23.4 | 35.2 |
| L3 | 1.0 | 1.2 | 3.3 | 7.1 | -9.7 | 16.2 |
| L4 | 2.0 | 2.3 | 2.6 | 11.7 | 0.15 | 5.3 |
| L5 | 5.0 | 4.9 | 3.4 | 5.4 | -7.9 | 14.7 |
| L6 | 10 | 8.8 | 2.9 | 9.7 | -14.8 | 40.5 |
| L7 | 15 | 14.1 | 3.6 | 7.8 | -7.8 | 15.0 |
| L8 | 20 | 18.4 | 3.2 | 7.0 | -9.4 | 15.7 |
| L9 | 30 | 27.6 | 4.7 | 5.5 | -8.9 | 18.2 |
| L10 | 40 | 36.2 | 3.8 | 4.0 | -7.9 | 17.9 |

**Table S2.** Inter-laboratory comparison study results for bovine plasma progesterone: range-based bias and average bias.

| Increasing concentration  groups (1–5) | Serum progesterone (ng/mL) | | Range-based Bias (Bias)%_RB_ (95%CI) |
| --- | --- | --- | --- |
|  | UF | TN |  |
| Group 1 | 0.28  0.73  0.80  1.30  1.43  1.53  1.86  1.91 | 0.20  0.45  0.72  1.09  1.29  1.22  1.49  2.08 | 19.1 (-13.1 to 51.4) |
| Group 2 | 2.04  2.17  2.49  2.83  2.89  3.00  3.09  3.27 | 1.84  1.80  2.24  2.25  2.75  3.24  3.49  2.91 | 7.3 (-16.5 to 31.1) |
| Group 3 | 3.91  4.13  4.61  4.69  4.70  5.01  5.06  5.14 | 4.05  3.97  4.19  4.81  4.11  4.71  4.24  5.36 | 5.0 (-11.0 to 20.9) |
| Group 4 | 5.17  5.61  6.26  6.61  7.48  8.41  9.01  9.37 | 4.76  5.00  5.37  6.32  6.52  8.46  7.75  8.80 | 9.2 (-1.9 to 20.3) |
| Group 5 | 10.8  11.2  11.65  13.70  14.85  16.10  18.68  28.04 | 10.2  10.4  10.1  12.5  16.2  13.8  16.1  25.8 | 8.3 (-7.0 to 23.6) |
| Average bias (Bias_AB_)% | 9.9 | | |

The 40 plasma samples for the between-laboratory comparison study were chosen as 5 clustering sets of 8 samples to investigate the range-based bias; the availability of samples also

influenced the selection. Samples were tested at UF with the IMMULITE® 2000 XPi P4 immunoassay (Siemens Healthineers Inc., Cary, NC), frozen at −80°C for <2 wk, and then sent overnight to Tennessee (TN) to be tested in one batch with the IMMULITE® 2000 XPi P4 immunoassay at the TN laboratory.

**Figure S1.** Bland-Altman mean difference plot between the plasma progesterone concentration ([P4]) measured by IMMULITE® 2000 XPi P4 immunoassay at the University of Florida (UF) and at the University of Tennessee (TN). The horizontal solid black line represents the mean bias, and the horizontal dashed orange lines represent the 95% limits of agreement. The horizontal dashed blue line represents the line of identity.


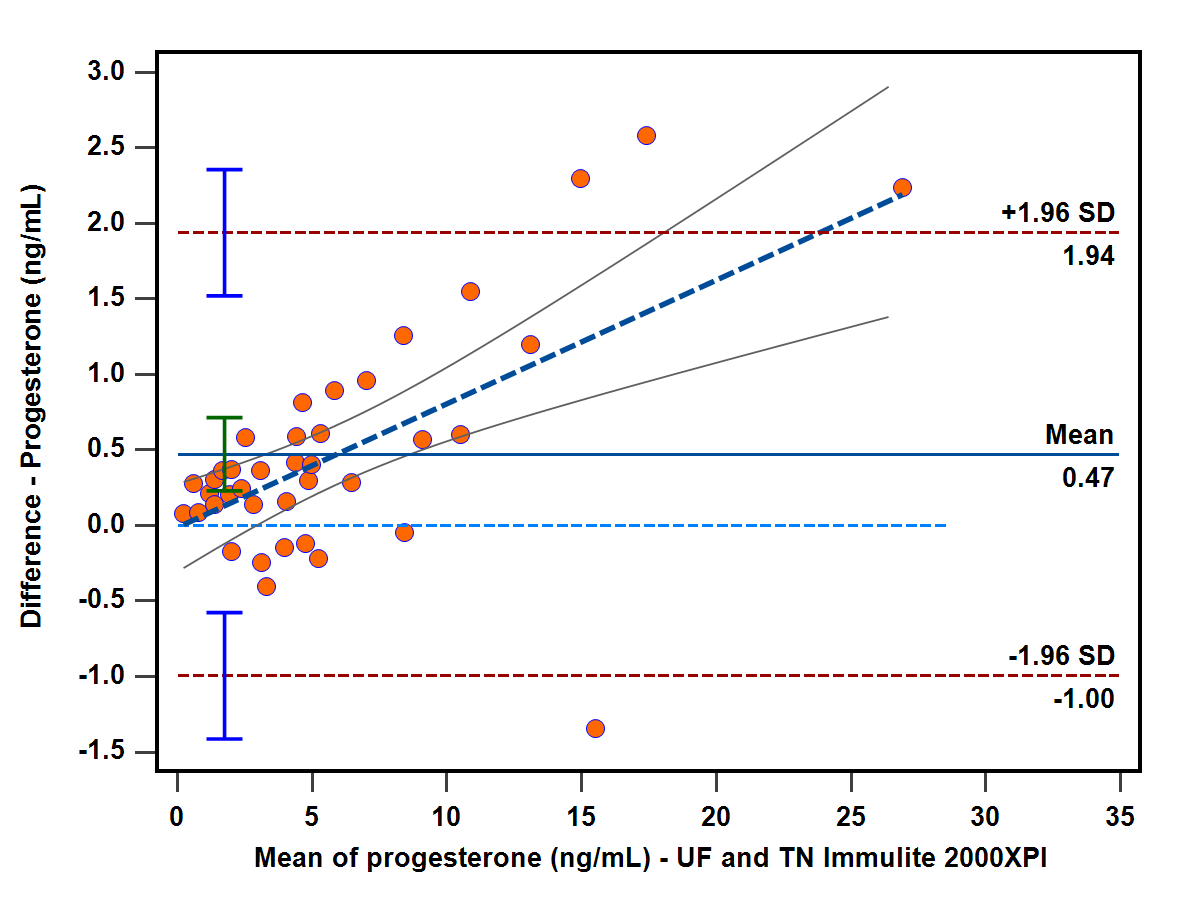

Supplement: Supplementary file 1 [file Data_Sheet_1.docx]
